# Neutrophil biology: an update

**DOI:** 10.17179/excli2015-102

**Published:** 2015-02-10

**Authors:** Yoshiro Kobayashi

**Affiliations:** 1 Division of Molecular Medicine, Dept. of Biomolecular Science, Faculty of Science, Toho University, Chiba, Japan

**Keywords:** neutrophil extracellular traps, neutropenia, degranulation, neutrophil subpopulations

## Abstract

Neutrophil extracellular traps (NETs) are involved in bacterial killing as well as autoimmunity, because NETs contain proteases, bactericidal peptides, DNA and ribonucleoprotein. NETs are formed via a novel type of cell death called NETosis. NETosis is distinct from apoptosis, but it resembles necrosis in that both membranes are not intact so that they allow intracellular proteins to leak outside of the cells. Removal of NETs and neutrophils undergoing NETosis by phagocytes and its subsequent response are not completely clarified, as compared with the response after removal of either apoptotic or necrotic neutrophils by phagocytes. How neutrophil density in peripheral blood is kept within a certain range is important for health and disease. Although the studies on severe congenital neutropenia and benign ethnic neutropenia have provided unbiased views on it, the studies are rather limited to human neutropenia, and mice with a mutation of mouse counterpart gene often fail to exhibit neutropenia. Degranulation plays a critical role in bactericidal action. The recent studies revealed that it is also involved in immunomodulation, pain control and estrous cycle control. N1 and N2 are representative of neutrophil subpopulations. The dichotomy holds true in patients or mice with severe trauma or cancer, providing the basis of differential roles of neutrophils in diseases.

## NETs Formation, NETosis and Removal of NETs

Neutrophil biology has been studied extensively in recent years. Among the topics of neutrophil biology, NETs have been drawing much attention as a new critical player in a variety of diseases, including infectious diseases and autoimmune diseases. 

NETs are web-like structures composed of chromatin, granular and cytoplasmic proteins. Brinkmann et al. are the first to describe NETs (Brinkmann et al., 2004[6]), and they suggested that NETs are actively released from living neutrophils. However, Brinkmann et al. showed later by using live cell imaging that NETs are formed at the final step of a program of active cell death called NETosis (Fuchs et al., 2007[14]). NETs are also formed without cell death under a certain condition (Yousefi et al., 2009[48]; Pilsczek et al., 2010[32]). The readers are advised to refer to excellent reviews on NETs (for instance, Kaplan and Radic, 2012[19]; Yipp and Kubes, 2013[47]; Marinod and Wagner, 2014[28]). 

### NETs formation

**Figure 1:**
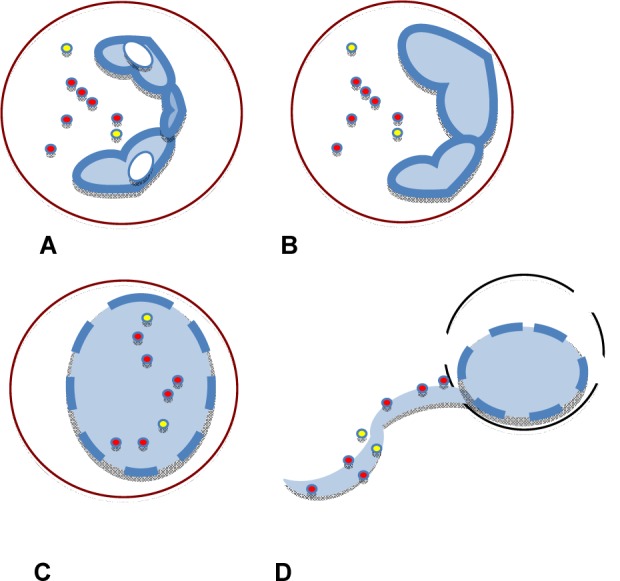
Upon stimulation with PMA or bacteria, neutrophils (A) undergo chromatin decondensation (B), disintegration of nuclear membrane and mixture of nuclear and cytoplasmic/granular materials (C), and cell rupture and release of NETs (D).

NETs formation is induced by PMA and a variety of microbes including *S. aureus* and *C. albicans *(Kaplan and Radic, 2012[19]) (Figure 1(Fig. 1))*.*

It has been reported that NETs formation depends on the generation of reactive oxygen species (ROS) by NADPH oxidase (Fuchs et al., 2007[14]). In support of the role of ROS in NETs formation, humanized sickle cell disease mice were found to contain plasma heme to cause NETs formation in a ROS-dependent manner upon TNF-α infusion (Chen et al., 2014[8]). Sickle cell disease is an inherited autosomal recessive disorder with a single amino acid substitution in the β chain of hemoglobin. The abnormal shape of red blood cells due to the mutation renders the patients prone to hemolysis, leading to the release of an excess amount of heme and thereby ROS in the circulation. 

It has also been reported that peptidylarginine deiminase (PAD) is involved in NETs formation. PAD converts arginine to citrulline that lacks arginine's positive charge (Nakashima et al., 1999[30]). Among several members of PAD family, PAD4 is an abundant component of cytoplasmic granules in neutrophils and eosinophils and its activation leads to widespread histone deimination (Asaga et al., 2001[2]). Histone hypercitrullination is detected on highly decondensed chromatin in HL-60 granulocytes and blood neutrophils. The inhibition of PAD4 with Cl-amidine decreases histone hypercitrullination and the formation of NET-like structures, whereas PAD4 treatment of HL-60 cells facilitates these processes (Wang et al., 2009[44]). Unlike PAD4^+/+^ mouse peripheral blood neutrophils, PAD4^-/-^ mouse peripheral blood neutrophils cannot form NETs after priming with chemokine, IL-8, followed by incubation with the pathogenic bacteria strain *S. flexneri* (Li et al., 2010[25]).

NETs are formed in only a fraction of neutrophils (for example, Fuchs et al., 2007[14]). They suggested the possibility that there might be a mechanism determining which neutrophil undergoes NETosis, although there may be another possibility that only a subpopulation of neutrophils can form NETs. 

Neutrophils sensed microbe size and selectively released NETs in response to large pathogens, such as *C. albicans* hyphae and extracellular aggregates of *M. bovis*, but not in response to small yeast or single bacteria (Branzk et al., 2014[5]). Why the authors failed to detect NETs formation in response to single bacteria is unknown at present. 

### NETosis vs. apoptosis and necrosis

NETosis is distinct from apoptosis for the reasons described below. NETosis is associated with disintegration of the nuclear envelope and mixing of nuclear and cytoplasmic material, loss of internal membranes, and disappearance of cytoplasmic organelles (Fuchs et al., 2007[14]). On the other hand, DNA fragmentation, phosphatidylserine exposure, and caspase activation, the hallmarks of apoptosis, are not associated with NETosis (Fuchs et al., 2007[14]). 

NETosis resembles necrosis in that both membranes are not intact so that they allow intracellular proteins to leak outside the cells. Some intracellular proteins such as myeloperoxidase, S100A8 and S100A9 remain associated with DNA after NETosis (Khandpur et al., 2013[21]), whereas others such as HMGB-1 and heat shock proteins do not (Khandpur et al., 2013[21]; Kaczmarek et al., 2013[18]). Therefore there is a possibility that, in both NETosis and necrosis, damage-associated molecular patterns (DAMPs) such as HMGB-1 and heat shock proteins are released to induce inflammatory responses. 

### Removal of NETs and its subsequent response

NETs are phagocytosed by dendritic cells (DCs). When NETs were loaded into myeloid DCs, and then the myeloid DCs were injected into naïve mice, they induced anti-neutrophil cytoplasmic antibody (ANCA). ANCA production was prevented by the treatment of NETs with DNase (Sangaletti et al., 2012[35]). Although there is a report that mere treatment of DCs with NETs is not sufficient to break immune tolerance (Liu et al., 2012[26]), NETs may be immunogenic under a certain condition. It is not known, however, whether or not DAMPs are involved in this process.

Human monocyte-derived macrophages are able to engulf NETs (Farrera and Fadeel, 2013[12]). In this study NETs were separated from neutrophils undergoing NETosis. Interestingly, C1q opsonized NETs, facilitating NETs clearance by the macrophages. Since uptake of NETs alone did not induce pro-inflammatory cytokine secretion, the authors claimed that the engulfment occurs in an immunologically silent manner. However, LPS-induced production of IL-1β, IL-6, and TNF-α was promoted by the uptake of NETs in contrast to the suppressive effect of apoptotic cells on LPS-induced cytokine production (Fadok et al., 1998[11]). Ingestion of apoptotic cells by macrophages induces production of anti-inflammatory cytokine, TGF-β (Fadok et al., 1998[11]), although this is not always the case (Kobayashi, 2011[23]). In this regard, it should be noted that nitric oxide is produced upon encounter of macrophages with apoptotic cells, thereby down-modulating neutrophil-specific chemokine (MIP-2) production *in vitro* as well as *in vivo* (Shibata et al., 2007[38]). 

NETs are degraded by serum DNase 1 (Hakkim et al., 2010[16]), although there is a report that serum DNase 1 is not able to degrade NETs at a physiological concentration (Farrera and Fadeel, 2013[12]). The reason for this discrepancy is not known at present, but the authors suggest the possibility that this is caused by the difference of the detection methods and preparation of test samples. Fluorescent assay (PicoGreen) was used to detect degraded dsDNA in the supernatant in Hakkim's study, whereas agarose gel electrophoresis was used to detect degraded DNA in Farrera's study. 

There is no report, however, which examines whether or not neutrophils undergoing NETosis are phagocytosed by macrophages and DCs.

### NETs in resolution

Aggregated NETs play a role in resolution of inflammation (Schauer et al., 2014[37]). In gout, monosodium urate (MSU) crystals accumulate neutrophils, but the inflammation resolves spontaneously within a few days, although MSU crystals still remain. Interestingly, aggregated NETs were found in gout patients, and such aggregated NETs have been called Tophi. If neutrophils are present in high density, MSU crystals can induce NETosis and aggregation of NETs. The aggregated NETs appear to promote the resolution of inflammation by degrading cytokines and chemokines via proteases in NETs. In individuals with impaired NETosis due to mutations in NADPH oxidase complex, MSU crystals induce uncontrolled production of inflammatory mediators from neutrophils and persistent inflammation. Furthermore, in models of neutrophilic inflammation, such as gout and zymosan-induced paw inflammation, NETosis-deficient mice due to a mutation of a regulatory subunit of the Nox2 complex develop exacerbated and chronic disease that can be reduced by adoptive transfer of aggregated NETs.

### NETs and diseases

NETs contribute to the lung pathology in a mouse model of acute sickle cell disease in which TNF-α was infused into mice (Chen et al., 2014[8]). The lung pathology was decreased by DNase I injection. 

NETs contribute to bacterial infectious diseases. In a mouse infectious disease model of necrotizing fasciitis with M1 Δ*Sda1 *(extracellular DNase) GAS strain, PAD4^-/-^ mice are more susceptible to infection with the bacteria than PAD4^+/+^ mice due to a lack of NET formation (Li et al., 2010[25]). 

NETs also contribute to autoimmune diseases, such as rheumatoid arthritis (RA) and SLE. Genetic polymorphisms were identified that enhance the expression of PAD4 and constitute a risk factor for RA (Suzuki et al., 2003[42]). A link between another autoimmune disease, SLE, and NETs formation has also been recently reported. TNF-α and IFN-α prime cells for NETs formation in response to anti-PR3, anti ribonucleoprotein, anti-HNP, or anti-LL-37 autoantibodies (Garcia-Romo et al., 2011[15]; Lande et al., 2011[24]; Kessenbrock et al., 2009[20]). High levels of inflammatory cytokines in autoimmune patients sensitize neutrophils to NETosis, whereas autoantibodies trigger a switch from apoptosis to NETosis.

## Neutropenia

The density of neutrophils in peripheral blood is regulated by production, egress and life span. Although one may suppose that G-CSF is a main player for such regulation via production, accumulating evidence indicates that this is not the case. To obtain unbiased views on the mechanism underlying the regulation, the investigation on genetic causes of neutropenia would be best suited. However, it has been rather limited to human studies. Moreover researchers often reported that mice failed to exhibit neutropenia upon introduction of a mutation of human gene into mouse counterpart and suggested the effect of background genes. Therefore mouse forward genetics approach should provide a unique finding in this research area. 

### Severe congenital neutropenia

There are several genetic defects causing congenital neutropenia (Klein, 2011[22]). Deficiency of the mitochondrial proteins HAX1 and AK2 causes premature apoptosis of myeloid progenitor cells associated with dissipation of the mitochondrial membrane potential, whereas mutations in elastase (ELA2/ELANE) and G6PC3 are associated with signs of increased endoplasmic reticulum stress. Mutations in the transcriptional repressor GFI1 and the cytoskeletal regulator WASP also lead to defective neutrophil production. It should be noted that *N*-ethyl *N*-nitrosourea mutagenesis yielded neutropenic mice with a mutation in GFI1 (Jaeger et al., 2012[17]).

Recently, mutations in the* JAGN1* gene were identified in severe congenital neutropenic patients, in which *ELANE, HAX1 and G6PC3* had no mutations (Boztug et al., 2014[4]). The ER in granulocytes from *JAGN1*-mutant patients is enlarged and granules are almost absent. N-glycosylation in the granulocytes is aberrant. A higher percentage of granulocytes from *JAGN1*-mutant patients undergo apoptosis as compared with granulocytes from healthy individuals. It should be noted that *Jagn1*-deficient mice do not show neutropenia.

### Benign ethnic neutropenia

Individuals of African descent and some ethnic groups in the Middle East have, as a group, lower neutrophil counts. This condition does not predispose individuals to infections and has therefore been termed benign ethnic neutropenia. Benign ethnic neutropenia represents the most common variant of low neutrophil counts. Genetic studies in people of African descent have highlighted the role of a polymorphism in the gene encoding the Duffy antigen receptor for chemokines (DARC) (Reich et al., 2009[33]). The Duffy null polymorphism (SNPrs2814778) is associated with the phenotype of ethnic neutropenia as well as protection against *Plasmodium vivax* malaria, but the molecular mechanism remains elusive. 

## Degranulation

Neutrophils undergo degranulation upon activation with stimuli, such as chemokines, to exert effector function as well as immunomodulatory function. There are four types of granules in neutrophils, azurophilic (or primary) granules, specific (or secondary) granules, gelatinase (or tertiary) granules, and secretory vesicles. Each granule contains a characteristic type of proteins, such as myeloperoxidase in azurophilic granules. Granular contents except for secretary granules are formed during neutrophil maturation, and primary, secondary and tertiary granules are formed in this order. Secretory granules, on the other hand, are formed through endocytosis in the end stages of neutrophil maturation, and therefore they contain plasma-derived proteins such as albumin. Among these granules, azurophilic granules are the most difficult to mobilize, followed by specific granules, gelatinase granules, and finally, secretory vesicles (Amulic et al., 2012[1]). Extensive analysis of granular contents has clarified how each granule is unique in terms of granular contents (Rørvig et al., 2013[34]).

In inflammation, infiltration of monocytes into the site is often caused by granule proteins released from neutrophils. There are several papers showing the involvement of PR3, LL-37, azurocidin (HBP/CAP37), and CG (Chertov et al., 1997[9]; Yang et al., 2000[46]; Soehnlein et al., 2008[40], 2009[41][39]) in induction of extravasation of monocytes. 

It has been reported that pain is controlled by opioid peptides secreted from granules (Machelska and Stein, 2006[27]). In post-sternotomy wounds of patients undergoing cardiac surgery, higher levels of opioid peptide expression was detected in neutrophils that were activated *in vivo* under the condition rich in IL-4 and IL-10 (Awad et al., 2012[3]). Therefore such opioids are likely released in the wound environment by the infiltrating neutrophils and contribute to peripheral analgesia at the wound-site. 

We also recently reported that estrous cycle is controlled by opioid peptides secreted from neutrophils in the ovary (Sasaki et al., 2011[36]). Proopiomelanocortin, a precursor of opioid peptides, was detected in a fraction of mouse peripheral blood neutrophils by immunohistochemical staining (Sasaki et al., 2011[36]), although it is not known at present whether or not this is caused by differential expression in neutrophil subpopulations.

## Neutrophil Subpopulations

### N1 vs. N2

Like M1 vs. M2 in macrophages, neutrophils are grouped into either N1 or N2, the former being pro-inflammatory, whereas the latter being anti-inflammatory. Suzuki and co-workers were the first to report neutrophil subpopulations, N1 and N2, by using a burn injury model (Tsuda et al., 2004[43]). N1 was induced under a mild burn injury, whereas N2 was induced under a severe burn injury. N1 was CD49d^+^, CD11b^-^, whereas N2 was CD49d^-^, CD11b^+^. 

Because severe trauma renders patients susceptible to infection, a burn injury model was then used for studying infection following trauma (Neely et al., 2014[31]). Burn injury caused systemic infection after subcutaneous infection of a wild type *P. aeruginosa*. Such mice showed a significant elevation in serum IL-10 and polarization of neutrophils into an N2 phenotype (IL-10^+^ IL-12^-^) in the spleen. The surface phenotype of N2 was Gr1^+^, CD11b^+^. Administration of a TLR5 agonist, flagellin, after burn injury, on the other hand, restored the neutrophil response towards a N1 phenotype, resulting in an increased clearance of a wild type strain of *P. aeruginosa*.

### Tumor-associated neutrophils (TAN)

Tumors are associated with not only macrophages but also neutrophils. 

TGF-β blockade significantly slows tumor growth through an influx of TANs (CD11b^+^/Ly6G^+^ cells) via neutrophil-attracting chemokines. TANs are more cytotoxic to tumor cells, and express higher levels of pro-inflammatory cytokines (N1) (Fridlender et al., 2009[13]). On the other hand, TANs infiltrating the tumor are driven by TGF-β to acquire a N2 pro-tumoral phenotype.

### Other examples of human neutrophil subpopulations

CD177, a GPI-anchored neutrophil antigen, is present in 0-100 % of neutrophils, but the fraction of CD177-positive neutrophils appears to be constant in each individual (Caruccio et al., 2004[7]; Moritz et al., 2010[29]). CD177 is localized primarily to the membrane of specific granules and to the plasma membrane. Its expression is determined by polymorphisms of the gene. 

Olfactomedin 4 (OLFM4), a specific granule protein, is present in roughly 20-25 % of neutrophils. Its presence is not regulated at the level of transcription (Clemmensen et al., 2012[10]). Both OLFM4-positive and -negative neutrophil subpopulations undergo apoptosis and phagocytose bacteria similarly. Both subpopulations were recruited equally to inflammatory sites *in vivo*. Only limited OLFM4 release was seen upon *in vivo* transmigration, and degranulation *in vitro* required strong secretagogues, namely cytochalasin B and ionomycin. However, OLFM4 was released upon NETs formation in response to PMA. It was detected in only a fraction of the NETs (Welin et al., 2013[45]).

The relation of these subpopulations with N1 and N2, however, is not known at present. The researchers have not examined cytokine production in these subpopulations.
